# The Impact of Metallic Nanoparticles on Stem Cell Proliferation and Differentiation

**DOI:** 10.3390/nano8100761

**Published:** 2018-09-26

**Authors:** Ahmed Abdal Dayem, Soo Bin Lee, Ssang-Goo Cho

**Affiliations:** Department of Stem Cell and Regenerative Biotechnology, Incurable Disease Animal Model & Stem Cell Institute (IDASI), Konkuk University, Seoul 05029, Korea; ahmed_morsy86@yahoo.com (A.A.D.); soobineey@naver.com (S.B.L.)

**Keywords:** metallic nanoparticles, stem cells, differentiation, proliferation, toxicity, regenerative medicine

## Abstract

Nanotechnology has a wide range of medical and industrial applications. The impact of metallic nanoparticles (NPs) on the proliferation and differentiation of normal, cancer, and stem cells is well-studied. The preparation of NPs, along with their physicochemical properties, is related to their biological function. Interestingly, various mechanisms are implicated in metallic NP-induced cellular proliferation and differentiation, such as modulation of signaling pathways, generation of reactive oxygen species, and regulation of various transcription factors. In this review, we will shed light on the biomedical application of metallic NPs and the interaction between NPs and the cellular components. The in vitro and in vivo influence of metallic NPs on stem cell differentiation and proliferation, as well as the mechanisms behind potential toxicity, will be explored. A better understanding of the limitations related to the application of metallic NPs on stem cell proliferation and differentiation will afford clues for optimal design and preparation of metallic NPs for the modulation of stem cell functions and for clinical application in regenerative medicine.

## 1. Introduction

**Figure 1:**
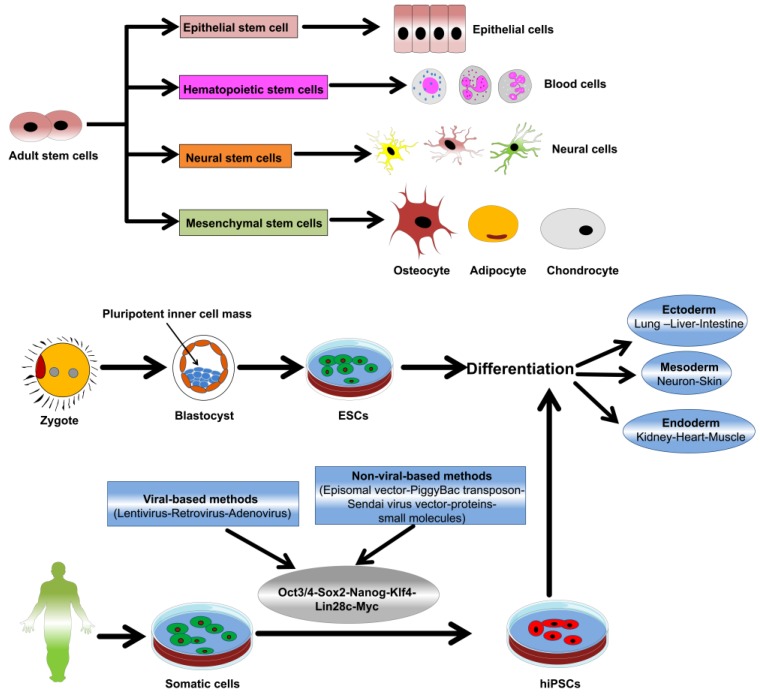
Representative diagram depicting the main types and sources of stem cells and their potential to differentiate into various lineages.

Stem cells possess unique properties, such as self-renewal and the ability to differentiate into multiple lineages based on the specific lineage inducer, which can be utilized for disease therapy [1]. As shown in Figure 1, stem cells are classified into embryonic stem cells (ESCs), adult stem cells (ASCs), and induced pluripotent stem cells (iPSCs).

ESCs are obtained from the inner cell mass of the growing blastocyst and have the potential to differentiate into ectoderm, endoderm, and mesoderm. At present, ethical issues and immune stimulation limit the application of ESCs in the regenerative medicine (Figure 1). The iPSC technology is based on the reprogramming of the patient-derived somatic cells through delivery of key transcription factors, namely Oct3/4, Sox2, c-myc, Nanog, and Lin28 via both viral and non-viral transduction methods (Figure 1). This approach provides similar pluripotency, gene expression profiles, epigenetics, and differentiation potential as that obtained from ESCs, while minimizing the ethical issues and immune activation [2]. Therefore, iPSCs represent a powerful tool for tissue regeneration.

On the other hand, ASCs, such as mesenchymal stem cells (MSCs), can be easily isolated from a variety of body organs and tissues, such as fat tissue, bone marrow, skin, brain, and bone. ASCs possess self-renewal properties and the capacity to differentiate into several lineages that emulate the original tissue or organ. In addition, the potency of MSCs to differentiate into bone, fat tissue, chondrocytes, and myocytes is evidenced, whereas their differentiation to other lineages, such as neurons, cardiomyocytes, and hepatocytes, is still debatable [3]. However, the self-renewal and differentiation capacities of MSCs are relatively weak in comparison to pluripotent stem cells, such as ESCs or iPSCs [4,5].

Generally, stem cell differentiation is laborious, as it requires formulation of various protocols and the addition of a wide spectrum of factors, undefined components, and chemicals that pose an economic burden, and their mechanism of action is not completely characterized. In this context, nanotechnology represents a powerful tool to overcome the drawbacks of the conventional method for promoting stem cell proliferation and differentiation, while improving the efficacy of stem cell differentiation and its applicability to regenerative medicine [6,7].

The vast scientific interest in the application of nanomaterials in stem cells is attributed to their easy preparation methods, biocompatibility, unique physical and chemical properties, and their efficient interaction with biomolecules. The applications of nanomaterials in stem cell tracking and therapy have been highlighted by various previous reports [8,9,10], and further studies on the relationship between nanomaterials and stem cell differentiation are needed.

Metallic nanoparticles (NPs), with a dimension of 1–100 nm, were first identified in 1857 by Michael Faraday who reported the presence of metallic NPs in an aqueous solution when he recognized the formation of ruby colored-AuNPs after a reaction of gold salt in aqueous solution [11]. Compared to the bulk material, metallic NPs possess a wide range of applications in biomedicine, which is attributed to their unique physicochemical characteristics, such as high energy atoms located on the particle surface area [12], high ratio of surface area-to-volume, high surface energy, surface plasmon resonances (SPR), presence of edges and corners, high dangling bonds, and electron storage capacity [13,14]. Metallic NPs with various sizes and shapes can be prepared with different methods including chemical, physical, and biological approaches [15]. Silver (Ag), gold (Au), and copper (Cu) represent pure metallic NPs, while zinc oxide (ZnO), titanium oxide (TiO_2_), cerium oxide NPs (nanoceria), Silicate NPs (SiNPs), and iron oxide NPs (IONPs) are metal oxide NPs, which are well-known with biomedical and pharmaceutical applications. 

The presence of high energy on the surface of metallic NPs results in metal-metal aggregation and, therefore, synthesis of a stable colloidal solution of metallic NPs is challenging [16]. Accordingly, scientists have attempted to use stabilizing agents, such as polyacrylic acid, polyvinylpyrrolidone, and polyvinyl alcohol, which are adsorbed onto the surface of particles and form a layer that minimizes particle aggregation, and ultimately, enables synthesis of a stable solution of the metallic NPs [17]. 

A wide range of nanomaterials that possess various properties and functions have been discovered. The tiny size of nanomaterials allows their efficient interaction with the cell surface and various cellular components [18]. Drug delivery, diagnostics, imaging, cancer therapy, catalysis, and anti-microbial activities are among the well-known biological applications of nanomaterials [19,20,21].

In this review, we will present a brief overview of the biomedical applications of metallic NPs, as well as the association between the nanotechnology and stem cells and their downstream applications. In particular, the influence of metallic NPs on stem cell proliferation and differentiation and the possible mechanism behind these effects will be elucidated in detail. In addition, the negative effects and toxicity of metallic NPs on stem cells will also be discussed. The mode of action of metallic NPs in stem cell differentiation and proliferation is still obscure. Thus, better understanding of the link between metallic NPs and stem cells will pave the way for therapeutic applications of metallic NPs in regenerative medicine. 

## 2. Biomedical Application of Metallic NPs

A number of published studies have highlighted the application of metallic NPs in medicine and disease therapy via various mechanisms. Here, we will briefly summarize the main biomedical applications and the possible mechanisms involved. 

### 2.1. Diagnostics

The application of nanomaterials in the diagnosis of disease has been shown enhanced the speed, sensitivity, and accuracy of the diagnostic process. NPs are considered efficient labeling materials due to their photochemical stability and the fact that their emission spectrum can be modulated [22]. The SPR of the metallic NPs, which permit light absorption and scattering at a certain wavelength, allow metallic NPs to serve as protein probes for the quantitative and qualitative measurement of the proteins. For instance, AgNPs and AuNPs are well-known examples of NP-based biosensors [23]. 

Metallic NPs can be suitable tools for the rapid analysis of proteins based on immunoassays. For example, research groups attempted to detect the tumor marker α-fetoprotein via the functionalization of AuNPs with an antibody targeting α-fetoprotein [24]. The binding of the antigen with the α-fetoprotein antibody-functionalized AuNP resulted in an increase in the size and hydrodynamic diameter of the bound AuNP, and ultimately, the concentration of the bound protein can be estimated [24]. Similarly, nucleic acid (60-mer DNA) and rabbit immunoglobulin (IgG)-functionalized AuNPs were applied for the estimation of DNA and protein at low concentrations using a lateral flow device [25]. Taken together, AuNP can be applied for fast clinical diagnosis.

The surface of metallic NPs could be functionalized with various biological recognition components, such as DNA, protein, peptides, aptamers, and specific receptors to produce nano-biosensors. These nano-biosensors provide an accurate and economically feasible method of analysis [26]. Interestingly, nano-biosensors can be applied to monitor cancer-related biomarkers, such as in chronic lymphocytic leukemia [27] and breast cancer [28]. 

NP-based imaging devices are commonly used for the diagnosis of complex diseases [29]. The potent absorption of the near infrared (NIR) by the metallic NPs allow them to serve as efficient contrast materials for in vivo imaging [30,31,32]. Metallic NPs can be applied as a contrast material with strong absorbance and scattering capacities for imaging devices such as computed tomography (CT) [33], surface-enhanced Raman scattering (SERS) [34], photoacoustic imaging (PAI) [35], magnetic resonance imaging (MRI) [36], and optical coherence tomography (OCT). For example, the tumor homing peptide, iRGD (CRGDK/RGPD/EC), was loaded with superparamagnetic iron oxide (SPIO) to label human pancreatic cancer cells [37]. In this study, human pancreatic cancer cells were seeded at 1 × 10^6^ cells/well, and after 24 h incubation, cells were washed three times with PBS and treated with two mL/well of a mixture of SPIO and iRGD peptides. The binding to the tumor cells were mediated via αvβ3 integrins. SPIO possessed high biocompatibility with iRGD, allowing for highly sensitive MR cancer imaging [37]. Additionally, sugar-coated paramagnetic Sugar/gadolinium-based AuNPs showed high capacity as MRI probes for cellular tracking [38]. The novel probes possessed high affinity for the carbohydrate-binding receptor at the surface of the cell due to the presence of sugar. These probes were considered to be efficient reporters to detect receptors in various cell lines at a high magnetic field [38].

Glioma cells at the inoculation site in brain tissue could be visualized using in vivo X-ray imaging when a high concentration of AuNPs was loaded along with the glioma cells. This imaging tool could shed the light on the occurrence of the angiogenesis, which was confirmed by measuring the leakage of AuNPs from cancer-related blood vessels [39]. Details regarding the possible applications of metallic NPs as contrast materials in bio-imaging were reviewed previously [40,41]. 

### 2.2. Drug Delivery

When designing drug delivery vehicles, one must develop the vehicles to display minimal toxicity with sustained release of the delivered drug, while overcoming the immunological barriers, delivering the desired dosage of the drug to the target tissue or organ. 

Passive drug delivery by metallic NPs occurs mainly in cancer cells, which is attributed to the abnormal vasculature. On the other hand, active drug delivery occurs through specific carriers that encapsulate the drugs and ultimately target specific chemical moieties or receptors in the target cells through functionalization and modification of the surface of NPs with desired functional components, such as peptides, DNA, carbohydrates, vitamins, and antibodies [42]. Conjugation of polyethylene glycol to the surface of NPs was one of the key tools for surface modifications of the carriers. This conjugation diminished the phagocytosis and opsonization of NPs via formation of a protective barrier between the NPs and plasma proteins [43]. 

The AuNP represents a model metallic NP, showing high efficiency for delivering recombinant proteins, nucleic acids, and drug compounds to a target area while controlling the release of the delivered compounds [44]. As an anti-cancer strategy, the delivery of methotrexate, a well-known anti-cancer drug, was conducted via the binding of its carboxyl group with the surface of AuNP [45]. Another example of metallic NP-mediated drug delivery is the delivery of the doxorubicin (DOX) through its conjugation with the surface of AuNP via a poly (ethylene glycol) spacer through a pH-sensitive linkage [46]. This delivery model showed efficient delivery and release of DOX, particularly in the multidrug resistant cancer cell line MCF-7/ADR [46]. Additional details on the possible application of noble metallic NPs in drug delivery for cancer therapy were reviewed elsewhere [47].

### 2.3. Additional Therapeutic Activities

#### 2.3.1. Anti-Cancer Activity

The anti-cancer activity of noble metallic NPs has been demonstrated in a wide range of studies. As described in the previous section, metallic NP-mediated delivery of anti-cancer drugs was considered a primary anti-cancer strategy. Several studies showed the efficiency of metallic NPs against proliferation of several cancers with various molecular mechanisms. The anti-cancer effect of AgNPs against human colon cancer was shown to be mediated via the activation of p53-induced apoptotic changes [48]. Moreover, AgNP treatment suppressed the proliferation of mouse fibrosarcoma L929 cells and disrupted cell division, leading to morphological changes [49]. The generation of reactive oxygen species (ROS) was one of the key molecular mechanisms involved in the anti-cancer activity of AgNPs against a wide range of cancer cells [50,51,52]. The anti-cancer properties of AuNPs have been widely investigated in several research reports [53,54,55,56]. 

ZnO nanorods efficiently suppressed the proliferation of human alveolar adenocarcinoma A549 cells in a concentration-dependent manner [57]. ZnO nanorode induced high levels of cytotoxicity which were mediated by extensive oxidative stress, and ultimately, upregulation of caspase, bax/bcl-2, and p53 signaling cascades [57]. Likewise, ZnO NP-induced ROS generation was implicated in its anti-cancer effects in human cancer cells [58]. Interestingly, the selectivity of ZnO NPs in killing human myeloblastic leukemia cells, but not the normal peripheral blood cells has been shown [59]. This cytotoxic action was ascribed to the potency of ZnO NPs to generate ROS, as well as the induction of ultrasound-mediated lipid peroxidation [59].

The unique optical characteristics of copper sulfide (CuS) NPs have also been exploited as an anti-cancer strategy. Irradiation of the CuS NPs with NIR at 808 nm resulted in photothermal-mediated anti-cancer proliferation activity against HeLa cells in a dose-dependent manner [60]. The conjugation of TiO_2_ NPs with folic acid showed potent anti-cancer effects against HeLa cells [61].

#### 2.3.2. Anti-Microbial Activity

The emergence of antibiotic resistance in a wide range of microbes has prioritized the development of novel antimicrobial agents, and nanomedicine represents one promising alternative to antibiotics [62]. The anti-bacterial activity of AgNPs is well-known [63], as AgNPs are commonly applied in wound dressings and used in the coating of the medical devices [64]. The anti-bacterial activity of AgNPs against *Escherichia coli* (*E. coli*) was mediated through disturbing a bacterial respiratory chain enzyme, which ultimately disrupted cell division and membrane integrity [65]. Moreover, AgNPs, when combined with the antibiotic vancomycin, showed synergistic anti-bacterial activity against the multidrug-resistant bacteria strain, *Streptococcus mutans* [66].

The anti-bacterial capacity of ZnO NPs has been well-characterized in previous reports [59,67,68]. The potent anti-bacterial activity of ZnO NPs was attributed to ROS generation and the consequent increase of the hydroperoxides, which ultimately led to lipid peroxidation-induced bacterial cell death [69]. In addition, an interesting study delineated the correlation between ZnO NP and its anti-bacterial activity [70]. The anti-microbial activity of TiO_2_ NPs was also shown in various research reports [71,72,73]. The anti-microbial activity of TiO_2_ NPs was elevated when combined with gold in an Au/TiO_2_ nanocomposite, a finding which was attributed to the alteration in the surface charge of TiO_2_ NPs when conjugated with gold [74].

#### 2.3.3. Anti-Inflammatory Activity

Inflammation can be caused by various factors, such as immune system activation, exposure to chemical agents or infectious agents, and trauma or injury. Several reports revealed that NPs display potent anti-inflammatory capabilities. The anti-inflammatory effect of metallic NPs can be achieved via functionalization of the particle surface with immune-related agents. For instance, AuNP was functionalized using IgG to produce AuNP-IgG, and the intravenous injection of AuNP-IgG had anti-inflammatory effects in a rat model [75]. Moreover, the platinum NPs markedly ameliorated the lipopolysaccharide-mediated inflammatory changes in RAW 264.7 macrophages [76]. This anti-inflammatory activity was attributed to the potent anti-oxidant capacity of platinum NPs [76].

The capacity of AgNP to diminish the peritoneal adhesion-mediated inflammation was highlighted [77]. Therefore, AgNP serve as candidate metallic nanomaterials for ameliorating adhesions after the surgical operations. Silver was included in silver-sulfadiazine cream for burn treatments [78]. The in vitro and in vivo anti-inflammatory activity of biologically synthesized AgNP using fruit extract was evaluated using UVB-exposed HaCaT cells and carrageenan-mediated edema in a rat paw model, respectively [79]. AgNP showed potent anti-inflammatory activity through a significant decrease in cytokine production in UVB-exposed HaCaT cells, as well as in the rat paw model after the exposure to carrageenan [79]. Additional information on the anti-inflammatory activity of the metallic NPs were illustrated elsewhere [80]. 

Taken together, the anti-inflammatory potential of the metallic NPs were evidenced in various reports and this property emphasizes the application of these nanomaterials as regenerative medicine devices.

#### 2.3.4. Disease Therapy

Metallic NPs are also involved in disease therapy. For example, metallic NPs efficiently ameliorated the pathogenicity of metabolic diseases, such as diabetes. In this regard, biologically synthesized AuNPs showed potent in vivo anti-diabetic activity in a rat model of alloxan-induced diabetes [81]. In addition, the in vivo anti-diabetic activity of ZnO NPs against type I and II diabetes mellitus was reported [82]. Both ZnO NPs and AgNPs showed potent anti-diabetic activities in streptozotocin-induced diabetes in male albino rats [83].

The application of the metallic NPs in ophthalmic disease therapy has been shown in previous reports. ROS scavenging activity of nanoceria showed a protective action against ROS-induced degeneration of primary culture cells in rat retina [84]. Moreover, the in vivo protective activity of the nanoceria suppressed the degeneration of the photoreceptor cells, ultimately protecting from vision loss [84]. Therefore, nanoceria could be key metallic NPs in ophthalmic disease therapy. This finding can pave the way for the application of the nanoceria particles in the therapy of other diseases that are induced by high ROS production.

Moreover, SiNPs have been shown to efficiently treat corneal neovascularization and angiogenesis when injected into the corneal stroma in a rabbit model [85]. Corneal neovascularization is considered to be one of the reasons behind vision loss. The anti-angiogenesis activity of SiNPs via blocking of vascular endothelial growth factor (VEGF) expression was associated with the treatment of the corneal neovascularization [85]. In line with this finding, the anti-angiogenesis properties of the metallic NPs, such as TiO_2_ NPs, AuNPs, and SiNPs, showed therapeutic capacities against the neovascularization of the retina in animal models [86,87,88]. Taken together, the suppressive action of the metallic NPs to the angiogenesis could be exploited in therapies against other diseases in which angiogenesis is implicated. 

Metallic NPs are also involved in the therapy of the neurodegenerative diseases. In this regard, the link between nanoceria and the activation of the autophagic machinery and the clearance of protein aggregates was exploited in nanoceria-mediated neurodegenerative disease therapy [89,90]. Moreover, nanoceria showed in vivo protective activity against brain ischemia, which was mediated via suppression of ROS [91].

## 3. Nanomaterial-Stem Cell Communication

The detailed pathways and mechanisms of NP-stem cell communication must be fully understood to enable full exploitation of nanotechnology in stem cell therapies. Application of nanomaterials in stem cell culture system is achieved through various methods, such as direct addition to the culture media, coating of culture dishes, and conjugation of the nanomaterials with defined scaffold for 3D culture. The interaction of the nanomaterials with the cell membrane or intracellular components, as well as the ultimate modulation of a specific cellular signaling pathways by the internalized NPs, has been demonstrated in previous reports [92,93]. 

**Figure 2:**
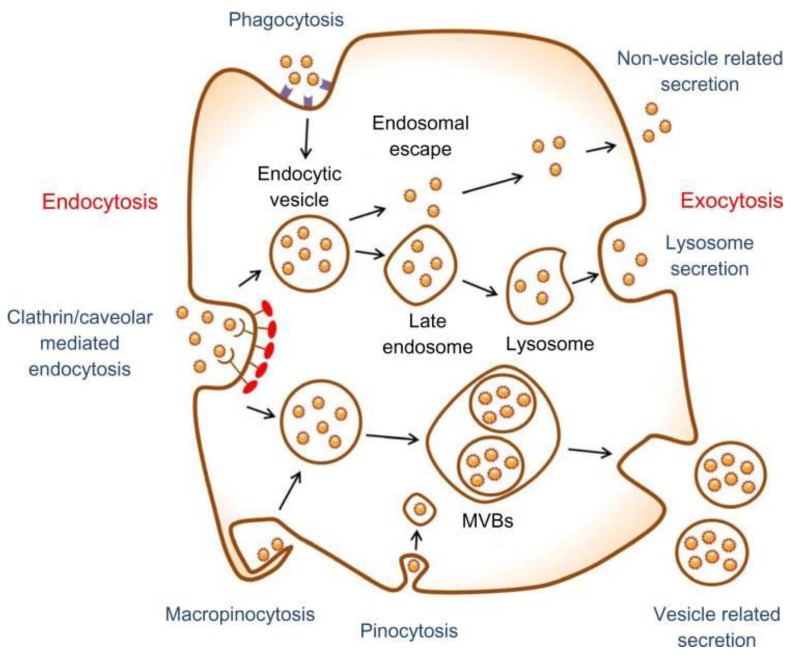
Schematic diagram describes the main pathways of NP uptake (endocytosis) and release (exocytosis) (Reproduced from [100] with permission from American Chemical Society).

Clathrin and caveolin-dependent endocytosis, phagocytosis, macropinocytosis, and pinocytosis represent possible mechanisms for the cellular internalization of NPs [94,95,96,97]. Clathrin or caveolin-dependent endocytosis is considered to be the main mechanism for the uptake of nano-size materials (Figure 2) [97,98]. The exocytosis or release of NPs is carried out via vesicle-dependent release, non-vesicle-dependent release, and lysosomal secretion. The cellular retention and release of particles can determine the extent of toxicity. There are several inhibitors modulating the mechanisms of cellular uptake, such as nocodazole, lovastatin, chlorpromazine, cytochalasin A, and genistein, which can be applied to characterize NP cellular internalization [99]. The detailed mechanisms and pathways of nanomaterials exocytosis have been explained and summarized elsewhere [95,99]. 

The size, shape, surface properties, stiffness, and the hydrophilic or hydrophobic properties of NPs are important for internalization into the cells [5]. For example, the uptake of NPs is inversely correlated with the particle size. In this context, higher uptake of smaller size NPs (30–50 nm) was reported, compared to bigger size NPs (50–200 nm), which show less cellular internalization [101,102]. Spherical-shaped NPs showed higher uptake rate than that that of non-spherical NPs [103]. Two-dimensional disk-shaped NPs possess less cellular uptake and a high potential to bind to the cell surface, compared to the spherical NPs that show high internalization [100]. Therefore, particle size and shape influence the biological function and toxicity of NPs and these factors should be considered during the design of nanomaterials.

The chemical modification of NPs by increasing the softness and the hydrophobicity has been shown to lead to a high rate of internalization [104]. Of note, a particle surface charge is implicated with cellular internalization rates. NPs possessing a positive charge can quickly enter the nucleus and avoid the lysosomal degradation, whereas particles with negative or neutral charges can easily localize to the lysosome instead of at the perinuclear region [105]. 

Preparation of engineered NPs with the desired functional group is one of the recent tools for modulation of cellular events for a particular biomedical application [106,107]. In this regard, a research group compared the internalization rate of polystyrene NPs and polystyrene NPs functionalized with an amine group in MSCs [108]. Amino-functionalized polystyrene NPs showed faster internalization and higher cellular uptake than that of unfunctionalized polystyrene NPs. Clathrin-dependent endocytosis was the main mechanism in the internalization of the amino-functionalized polystyrene NPs [108]. 

TiO_2_ nanorods functionalized with various functional groups, such as carboxyl groups (–COOH), poly (ethylene glycol) (–PEG), and amines (–NH_2_), showed a variation in their uptake by rat bone marrow-derived MSCs (rBM-MSCs) according to data with transmission electron microscopy (TEM) and inductively coupled plasma mass spectrometry (ICP-MS) [109]. The high rate of cellular internalization was detected in TiO_2_–NH_2_ nanorods and the core nanorods, compared with TiO_2_–COOH and TiO_2_–PEG nanorods that showed lower uptake. However, the TiO_2_ core nanorods possessed the most toxic effects via ROS generation, which could be mitigated by the addition of the surface functional groups [109]. Therefore, modification of the particle surface by the addition of functional group can modulate its cellular uptake and consequent toxicity. External stimuli such as tunable magnetic fields can influence the high rate of particle internalization into stem cells [110].

It is noteworthy that ROS modulation represents one of the key mechanisms of metallic NP-associated cellular functions [52]. In addition, ROS generation is implicated in the modulation of stem cell differentiation [52,111]. Collectively, particles with tailored physicochemical properties can exert differential influence on stem cell differentiation and proliferation.

## 4. Metallic NPs and Stem Cell Differentiation and Proliferation

Thus far, a significant scientific interest has been directed toward the application of nanomaterials in the modulation of stem cell proliferation and differentiation for further application in regenerative medicine. Here, we will elucidate the impact of the key metallic NPs on the differentiation and proliferation of stem cells with the possible mode of action and further applications in regenerative medicine.

### 4.1. AuNP

Due to their unique characteristics, biocompatibility, and low toxicity, AuNPs have been regarded as favorable materials for directing stem cell fate and tissue regeneration. AuNPs promoted the differentiation of mouse ESCs (mESCs) into dopaminergic (DA) neurons, which occurred as a result of AuNP-induced activation of the mTOR/p70S6K signaling pathway [112]. The nanocomposite of AuNP and the electrospun nanofiber scaffold showed an increase in the neurite length and axon elongation [113]. Therefore, this AuNP scaffold could be a promising device for the regeneration of damaged nerves. 

AuNP abrogated the oxidative stress-induced apoptotic changes in retinoic acid (RA)-exposed F9 teratocarcinoma stem cells [114]. Moreover, AuNP-treated F9 teratocarcinoma stem cells showed neuronal differentiation, which was evidenced by the upregulation of collagen type IV, RA binding protein, Gata 6, and laminin 1 [114]. 

There is great body of evidence showing the link between AuNPs and the osteogenic differentiation of stem cells, which is summarized elsewhere [115]. The shape, size, and surface characteristics of AuNPs impacted their potential to induce the osteogenic differentiation of MSCs [101]. Rod-shaped AuNP with a size of 70 nm markedly promoted the osteogenic differentiation, while 40 nm rod-shaped AuNPs suppressed osteogenic differentiation [101]. In human adipose-derived stem cells (hADSCs), the induction of osteogenic differentiation is prompted upon exposure to AuNPs sized 30 and 50 nm [102]. Photo-curable gelatin hydrogels loaded with AuNPs markedly enhanced the proliferation and osteogenic differentiation of hADSCs [116]. This effect was proved in vivo via the regeneration of bone defects. The Wnt/β-catenin, extracellular signal–regulated kinase (ERK), and p38 signaling pathways were revealed to be the main pathways involved in AuNP-induced osteogenic differentiation [117,118,119].

In human BM-MSC (hBM-MSCs) and MC3T3-E1 cells, miR029b-delivered polyethyleneimine (PEI)-capped AuNPs efficiently promoted the osteogenic differentiation with almost no toxicity [120]. This osteogenic differentiation-inducing capacity was evidenced through the upregulation of the osteogenic differentiation-related genes, namely alkaline phosphatase (ALP), osteopontin (OPN), osteocalcin (OCN), and Runt-related transcription factor 2 (RUNX2) [120]. The chirality of the surface of AuNPs through the anchoring of the chiral poly (acryloyl-l (d)-valine) (l (d)- PAV) to finally produce l(d)-PAV-AuNPs, which led to the promotion of the osteogenic differentiation of MSCs [121]. l(d)-PAV-AuNPs-exposed MSCs showed upregulation of osteogenic differentiation marker genes such as OCN and collagen type I (COL I), activation of mitogen-activated protein kinase (MAPK)/p38 pathway, and calcium mineralization [121]. 

One research team examined the effect of a small size of AuNPs (4 nm) on the differentiation of hBM-MSCs compared with large size AuNPs (40 nm). The small AuNPs markedly suppressed osteogenic differentiation, while promoting the adipogenic differentiation of hBM-MSCs [122]. This effect is ascribed to ROS production by the small size AuNPs that resulted in a differential differentiation effect. Taken together, ROS mechanism is implicated in differentiation modulation in small size AuNP-treated hBM-MSCs. 

AuNP-loaded functionalized nanofibrous scaffold promoted the cardiogenic differentiation of the MSCs, which was highlighted via morphological changes, formation of the contractile proteins, and the upregulation of the cardiogenic differentiation-related markers [123]. Similarly, AuNP-loaded bovine serum albumin (BSA)/Polyvinyl alcohol (PVA) nanofibrous scaffolds promoted the cardiomyogenic differentiation of BM-MSCs, which resulted in an increase in the cell proliferation, phenotypic changes, and upregulation in the cardiomyocyte-related protein markers [124]. Taken together, AuNP-loaded scaffolds can be applicable for regeneration of damaged heart muscles or cardiac infarction therapy.

An interesting research report showed a novel monitoring method of the differentiation potential of mouse neural stem cells (mNSCs) using 3D graphene oxide (GO)-encapsulated AuNPs [125]. This method is based on the enhancing of SERS signals with GO-encapsulated AuNPs, which vary according to the differentiation status that related to C=C bond and its degree of saturation. For instance, C=C bond is abundant and possesses a high degree of saturation in the undifferentiated state of NSCs. The correlation between the SERS signals based on the differentiation status was further confirmed with immunofluorescent staining using the confocal microscopy. This method could pave the way to design novel hybrids of graphene and other metallic NPs for the measurement of the differentiation status of stem cells.

### 4.2. AgNPs

**Figure 3:**
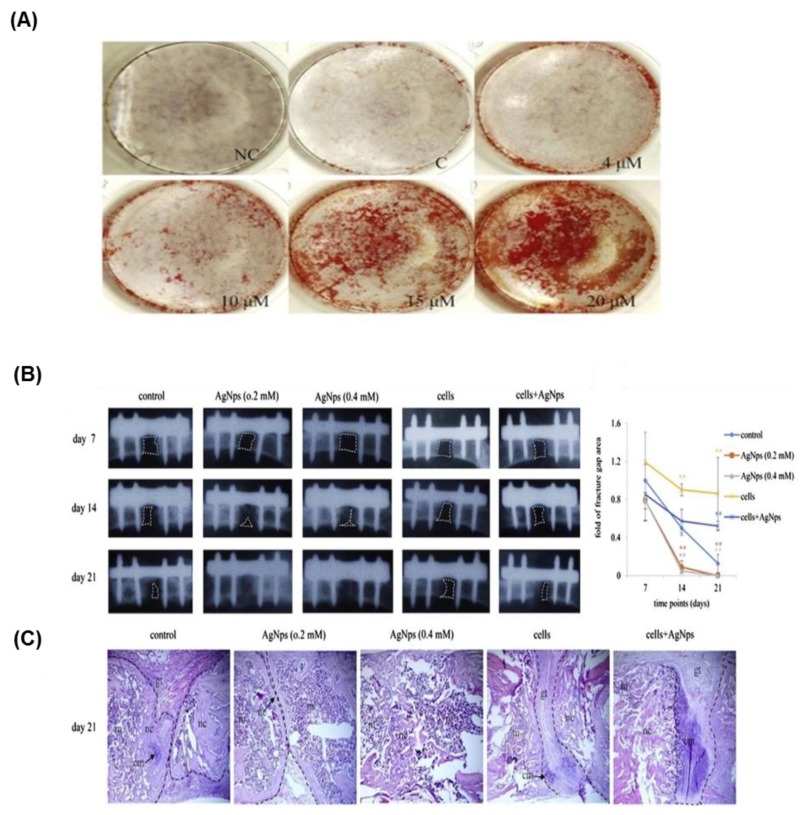
(**A**) AgNPs promoted the osteogenic differentiation of mMSCs in a dose-dependent manner as shown by alizarin red staining. (**B**) The healing of mouse bone fracture after the exposure to AgNPs is highlighted by the plain X-ray radiographic analysis of the location of fracture and the graphic data that represent the fracture gap closure. (**C**) Histological analysis of the fracture site in mice scarified after 21 days was carried out using hematoxylin and eosin staining of the middle section of the fracture. This figure was reproduced from Reference [105] with permission from Elsevier.

The anti-microbial capacity of AgNP is behind its wide-spread application in the biomedical field [126]. Regarding stem cell differentiation, previous reports have demonstrated both the positive and negative impacts of AgNPs on stem cell differentiation. AgNPs of size 10 or 20 nm [127] and of size 30 nm [128] showed no marked toxic action on the differentiation of MSCs [129]. The influence of AgNPs on promoting osteogenic differentiation of urine-derived stem cells has been investigated [130]. In this study, the toxicity of AgNPs was first screened in parallel with its bulk material, silver nitrate (AgNO_3_), for selection of non-toxic concentrations for the osteogenic differentiation. AgNO_3_ did not show any marked influence on osteogenic differentiation. Similarly, AgNPs enhanced osteogenesis in mouse MSCs (mMSCs) through the upregulation of transforming growth factor-beta (TGF-β) and bone morphogenic protein (BMP) signaling (Figure 3) [105]. This effect was confirmed using a femoral fracture mouse model, as AgNP promoted callus formation and the closure of bone fracture. 

The functionalization of AgNP with the photo-activated miR-148b mimic (miR-148b-AgNP construct) led to the osteogenic differentiation of the human autologous adipose derived mesenchymal stromal/stem cells (hASCs) [131]. AgNP successfully delivered this photo-activated miR-148b mimic into the intracellular space without the use of transfection vectors [131]. Upon photo-activation, this construct results in upregulation of osteogenic differentiation-related markers. 

AgNP, as a coating material, enhanced the adipogenic differentiation of hBM-MSCs with the activation of the adipogenic differentiation markers and the accumulation of fat droplets [132]. AgNP-induced ROS generation was implicated in the promotion of adipogenic differentiation of hBM-MSCs and AgNPs suppressed osteogenic differentiation [132]. AgNPs promoted the neuronal differentiation of F9 teratocarcinoma stem cells when treated with a low dose [133]. This differentiation was shown with the upregulation of the neuronal differentiation markers and suppression of stemness markers. AgNP-induced neuronal differentiation of F9 teratocarcinoma stem cells indicates that AgNPs could be a potent metallic nanomaterial for cancer stem cell (CSC) therapy.

### 4.3. TiO_2_

Owing to the unique mechanical and chemical properties and the biocompatibility of the titanium, it has become a well-known material in the prosthetic devices and dentistry [134,135]. A research report based on protein interaction network analysis emphasized the influence of TiO_2_ NPs on the promotion of the neuronal differentiation of the mNSCs, which was validated by showing the positive expression of a neural marker, βIII-tubulin, using immunofluorescent staining and fluorescence-activated cell sorting (FACS) analyses [136]. Interestingly, TiO_2_ NP-exposed cells showed upregulation in the phosphorylation and the expression level of gap junctional intercellular communication protein connexin-43.

The nanoscale geometry of various TiO_2_ nanotubes played important roles in the modulation of stem cell behavior and fate [137,138]. For instance, small-sized TiO_2_ nanotubes (15–30 nm) underpinned cell adhesion and spreading and therefore promoted the osteogenic differentiation of rat BM-MSCs (rBM-MSCs) [139]. Titanium nanotubes promoted the osteogenesis of pulp and adipose tissue-derived stem cells [140].

TiO_2_–NH_2_, TiO_2_–COOH, and TiO_2_–PEG did not show any negative effects on the adipogenic differentiation of rBM-MSCs [109]. The promotion of osteogenic differentiation of hBM-MSCs cultured on TiO_2_ surface was evaluated in comparison with cells grown on a coverglass [141]. Moreover, TiO_2_ surface-cultured cells showed high adhesion, which mediated the high level of phosphorylation of focal adhesion kinase (FAK).

One research group tested the impact of TiO_2_ nanotubes with various diameters on the osteogenic differentiation of hASCs [142]. TiO_2_ nanotube of size 70 nm was found to be ideal for induction of osteogenic differentiation of hASCs, which was mediated with the increase in the methylation of the histone H3 at lysine four in the promoter regions of the osteogenic differentiation-specific marker genes, namely OCN and RUNX2 [142]. This TiO_2_ nanotube-mediated osteogenic differentiation was attributed to the inhibition of demethylase retinoblastoma binding protein 2 (RBP2). Taken together, metallic NPs can exploit epigenetic mechanisms for the modulation of stem cell differentiation.

Using a hydrothermal method, a research group ornamented the surface and the inside of the titania nanotubes (TNTs) with TiO_2_ NPs of size 3–8 nm to form TNT-TiO_2_ and tested the antibacterial activity as well as compatibility with cultured stem cells [143]. Upon photo-induction with UV light exposure, prolonged wettability was enhanced with consequent potent antibacterial activity against *Porphyromonas gingivalis* and *Streptococcus mutans*. Moreover, TNT-TiO_2_ possessed a unique topographical surface and high energy, which ultimately enhanced stem cell osteogenic differentiation [143]. 

Another mechanistic study uncovered the role of mitogen-activated protein kinase kinase kinase 11 (MAP3K11), Na^+^/K^+^ transporting ATPases ATP1A2 (alpha 2 polypeptide), and ATP1A3 (alpha 3 polypeptide) in 100 nm TiO_2_ nanotube-induced osteogenic differentiation of bone marrow stromal cells [144]. Interestingly, the TiO_2_ nanotube could be a nano-reservoir for the loading of osteogenesis-promoting components, such as BMP2 [145]. In this study, TiO_2_ nanotubes coated with the multi-layered gelatin and chitosan enhanced the osteogenic differentiation of MSCs, which was attributed to the controlled release of BMP2.

### 4.4. IONPs

IONPs can be used for various biomedical applications, such as cancer therapy, small interference RNA (siRNA) delivery, bio-imaging, and stem cell tracking [146,147,148,149]. SPIO NPs are a type of IONPs that possess superparamagnetism property [150,151]. This superparamagnetism property enables SPIO NPs to have a magnetic property when subjected to an external magnetic field. Coating of SPIO with dextran (DEX) and then labeling of ESCs led to a marked induction of myogenic differentiation under pulsed electromagnetic field, which was evidenced through the upregulation of the expression level of the myogenic-specific markers, MyoG and Myh2 [152]. A recent study showed the potent in vivo therapeutic capacity of DEX-coated IONPs (DEX-IONPs) using a mouse model (male Balb-c nude mice) of Parkinson’s disease, which is induced by stereotactic injection (intrastriatal injection) of 6-hydroxydopamine (6-OHDA) [153]. 6-OHDA showed a significant loss of DA terminals mainly in the damaged striatum and a marked loss of cell bodies of DA cells in substantia nigra pars compacta (SNpc) of the damaged site. Three weeks after 6-OHDA lesion in mice, DEX-IONP-labeled hMSC (DEX-IONP-hMSCs) were intracerebrally injected into the right ventricle. DEX-IONP-hMSC-injected mice showed a partial behavioral recovery and high density of the positive immunofluorescent staining of tyrosine hydroxylase (TH) in 6-OHDA-lesioned striatum, indicating the migratory capacity of DEX-IONP-hMSCs toward the lesioned striatum. However, the control unlabeled hMSCs did not show a significant migration to the 6-OHDA-lesioned site. Moreover, the report showed the transdifferentiation of DEX-IONP-hMSCs into TH positive cells when localized into the damaged SNpc that ultimately replaced the damaged DA neuron. These in vivo results were confirmed in vitro using human neuroblastoma cell line SH-SY5Y model that retained the features of the DA neurons. Conditioned medium-derived DEX-IONP-labeled hMSC significantly recovered the 6-OHDA-induced damage of SH-SY5Y cell-derived DA neurons. Moreover, the coculture of hMSC/DEX-IONP-labeled hMSC and SH-SY5Y cells was carried out using a Transwell system. In this coculture system, SH-SY5Y cells were grown in the bottom chamber and subjected to the differentiation into DA neurons using specific culture medium for two weeks and hMSC/DEX-IONP-labeled hMSC was grown in the upper chamber of the Transwell system. The exposure of SH-SY5Y cells to 6-OHDA promoted the DEX-IONP-labeled hMSC, but not the control unlabeled hMSC to migrate to the 6-OHDA-damaged SH-SY5Y cells. Interestingly, this migratory effect was abolished after treatment of the inhibitors targeting chemo-attractants, namely, chemokine receptor type 4 (CXCR4) and epidermal growth factor receptor (EGFR) that assumed to recruit DEX-IONP-labeled hMSC to the site of damage [153]. Therefore, IONPs could be a powerful tool for the neurodegenerative disease therapy.

**Figure 4:**
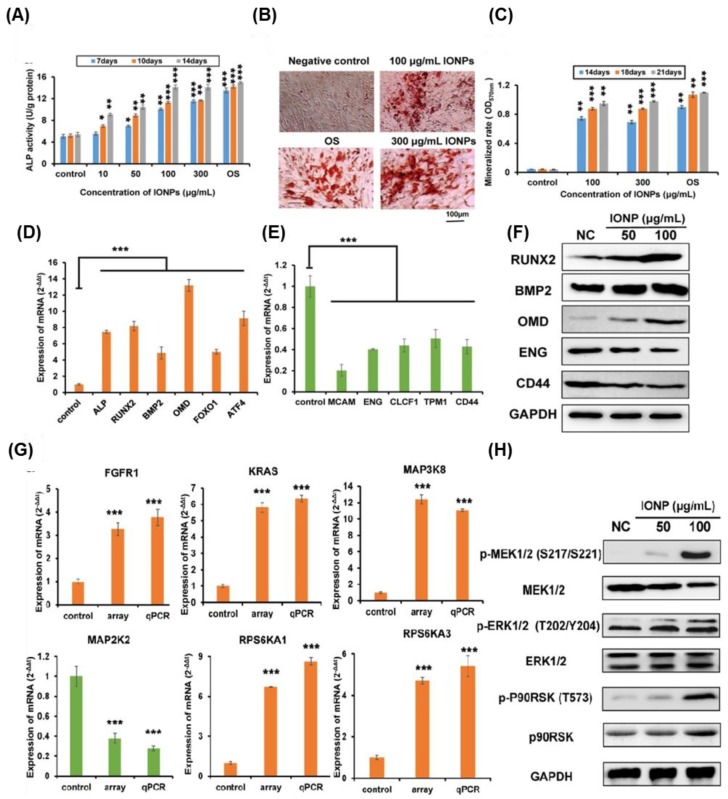
IONPs promoted the osteogenic differentiation of hBM-MSCs. (**A**) IONP-exposed hBM-MSCs showed high ALP activity in a dose-dependent manner (* *p* < 0.05; ** *p* < 0.01; *** *p* < 0.001). Scale bar: 100 μm. (**B**) Calcium mineralization indicated by alizarin red S staining. (**C**) Quantification of alizarin red S staining (** *p* < 0.01; *** *p* < 0.001). (**D**) The quantitative real-time PCR data showed the upregulation of the osteogenic differentiation-specific genes when exposed for one week to IONPs at 100 μg/mL (*** *p* < 0.001). (**E**) The downregulation of the MSC-specific markers after exposure to IONPs at 100 μg/mL for one week (*** *p* < 0.001). (**F**) Upregulation of the protein level of the osteogenesis-associated proteins in IONP-treated hBM-MSCs. (**G**) The quantitative real-time PCR analysis results displayed the significant increase in the expression levels of mRNAs of the classical MAPK-related genes upon exposure to IONPs (100 μg/mL) for one week (*** *p* < 0.001). (**H**) Western blot analysis showed the phosphorylation of MAPK-associated proteins. This figure is reproduced from [154] with Elsevier’s permission.

IONP-grown hBM-MSCs showed enhanced osteogenic differentiation in a dose-dependent manner (Figure 4) [154]. Gene microarray and bioinformatics analyses were carried out to identify the molecular mechanisms involved in IONP-induced osteogenic differentiation of hBM-MSCs, which uncovered the activation of the classical MAPK signal pathway in IONP-differentiated hBM-MSCs. For confirmation of the previous analyses, a quantitative real-time polymerase chain reaction (PCR) analysis was carried out to measure the expression level of MAPK-associated genes, such as FGFR1 (fibroblast growth factor receptor 1), MAP3K8 (Tpl2/Cot), KRAS (Kirsten rat sarcoma viral oncogene homolog), RPS6KA1 (ribosomal protein S6 kinase, 90 kDa, polypeptide 1), RPS6KA3, and MAP2K2 (MEK2). The significant upregulation of the expression level of the aforementioned genes was shown, which was in line with the microarray analysis (Figure 4). Additionally, Western blot analysis was employed to confirm the gene expression analysis that showed a marked phosphorylation of ERK1/2, MEK1/2, and p90RSK. 

The magnetic field-induced assembly (stripe-like) of magnetic IONPs was exploited for the conversion of primary mouse bone marrow cells into osteoblasts [155]. The interface between IONP magnetic assemblies and the cells is implicated in the induction of osteogenic differentiation rather than the particles internalization into the cells. Magnetic IONPs were shown to boost the expression level of the long noncoding RNA *INZEB2*, which ultimately promoted the osteogenic differentiation of hBM-MSCs [156]. In this context, *INZEB2* suppressed the expression of *ZEB2*, which is a key transcription factor implicated in the inhibition of BMP/Smad-associated activation of the transcription of the osteogenic differentiation. 

Stimulation of Fe_3_O_4_/BSA-loaded IONP with an external stimulus, such as a static magnetic field resulted in its high uptake rate into hBM-MSCs and a marked promotion of the osteogenic differentiation [110]. Coating of IONPs with human serum albumin (HSA) is one of the strategies that allow its binding to proteins. In this context, a study showed that IONPs coated with HSA (IONP/HSA) bound to FGF2 and promoted the multi-lineage differentiation of hBM-MSCs into bone cells, adipose tissue, and neurons [157].

Coculture of MSCs with cardiac cells led to the cardiac priming of MSCs and therefore enabled the application of MSCs in cardiac disease therapy. Accordingly, IONPs promoted potential crosstalk between the cardiomyoblasts and MSCs via the upregulation of the expression of a gap junction protein, namely connexin 43 in IONP-exposed cardiomyoblasts [158]. IONP-induced gap junction communication between cardiomyoblasts and MSCs resulted in the marked activation of electrophysiological cardiac markers and paracrine function related to the repair of the myocardial infarction. The therapeutic effect of the IONP-cocultured MSCs was proved in vivo using a myocardial infarction rat model [158].

SPIO NPs promoted the proliferation of hMSCs via abrogating the intracellular H_2_O_2_ and enhanced the progression of the cell cycle by upregulation of the cell cycle-related proteins, such as cyclin B, cyclin D1, and cyclin-dependent kinase 4 (CDK4) [159]. Therefore, SPIO NPs are considered as being a safe nanomaterial for stem cell labelling. 

### 4.5. Other Metallic NPs

Zinc is one of the most plentiful trace metals in the human body and was reported to be essential for the regeneration of bone. In this respect, the differentiation of hMSCs into osteoblasts was enhanced upon their culture with polymeric fibrous polyethersulfone-polyethylene glycol (PES-PEG) electrospun composites coated with willemite or Zn_2_SiO_4_ bioceramic NPs, which was evidenced by calcium mineralization, high ALP activity, and upregulation of the osteogenesis-related markers [160]. The topographical orientation of ZnO NPs on the surface had a significant impact on stem cell differentiation; cells grown on horizontal ZnO nanorods showed higher adhesion and survival rate compared with the cells cultured on vertical nanorods [161].

The high biocompatibility of silica-containing materials allows them to be favorable for application in bioengineering. SiNPs have been reported to promote the osteogenesis of hMSCs, which was indicated via the activation of ALP and formation of bone nodules [162]. SiNP of 50–120 nm stimulated the proliferation of hADSCs via the phosphorylation of ERK1/2 signaling [163]. SiNP-treated hMSCs showed high focal adhesion and upregulated expression of the connexin-43 [164]. This high expression level of connexin-43 enabled hMSCs to be co-cultured with cardiac myoblasts and ultimately aided the regeneration of infarcted cardiac tissue. The conjugation of SiNP with insulin markedly enhanced the adipogenic differentiation of rMSCs with minimal cytotoxicity [165]. 

Mesoporous SiNPs successfully delivered Nurr1 plasmid DNA and Rex1 siRNA into iPSCs within 6 h, which resulted in their neural differentiation [166]. Therefore, mesoporous SiNPs represent a non-viral delivery method that maintains the genetic integrity of iPSCs. Collectively, reports on the impact of SiNPs in stem cell differentiation are limited; therefore, further research in this field is required. A hybrid of SiNP-GO was prepared via the combination of the SiNPs (with a positive charge) with a GO nanosheet and its potential to promote the neural differentiation of hNSCs was examined [167]. SiNP-GO hybrid markedly promotes the survival rate of hNSCs (more than 3 weeks) and the neural differentiation that shown in the upregulation of the early stage (Tuj-1) and late stages (MAP2) neural markers as well as the axonal marker, growth associated protein 43 (GAP43). Moreover, it promoted the growth and the alignment of the axons, which enable SiNP-GO hybrid to be a promising hybrid material regeneration of the injured nerves. However, the mechanism of SiNP-GO-induced alignment of the differentiated hNSCs is still unclear and needs further investigations.

The incorporation of nanoceria into a hydroxyapatite coating led to the promotion of proliferation and osteogenic induction, which was attributed to the activation of BMP signaling [168]. In addition, nanoceria showed potent anti-inflammatory activity in RAW264.7 macrophages [168]. Nanoceria suppressed the adipogenic differentiation of rMSCs via the suppression of ROS-induced adipogenic differentiation induction [169]. Citrate-stabilized nanoceria significantly enhanced the proliferation of MSCs in a concentration-dependent manner [170]. 

The modification of the bone scaffold with nanoceria enhanced the proliferation of MSCs, which was mediated through the upregulation of the intracellular calcium level and the consequent increase of the expression level of the angiogenic factor, VEGF, which ultimately enhanced the vascularization of the bone grafts [171]. Nanoceria-exposed MSCs showed high expression of proliferation-associated genes and inhibition of apoptotic changes. Similarly, nanoceria improved the proliferation of human dental pulp-derived MSCs via modulation of proliferation-associated gene expression and cell cycle [172]. On the other hand, nanoceria suppressed differentiation of NSCs, which was shown by inhibition of the expression level of the neuron-related marker, βIII-tubulin and glial fibrillary acidic protein (GFAP) [173]. 

Barium titanate NPs coated with glycerol-chitosan enhanced the proliferation and the osteogenic and adipogenic differentiation of rMSCs even at high concentrations [174]. 

## 5. Metallic NPs and Stem Cell Toxicity

As detailed above, metallic NPs showed a wide range of positive effects on stem cell differentiation and have a number of applications in regenerative medicine. However, metallic NPs also have negative effects on stem cells; this should be considered during the application of the metallic NPs in stem cell research. AgNP-exposed human and rat-derived embryonic NSCs showed marked toxicity in a dose-dependent manner [175]. AgNP-induced neurotoxicity was ascribed to high ROS generation, mitochondrial dysfunction, activation of BAX protein, and the release of the lactate dehydrogenase [175]. This neurotoxic effect was abolished upon treatment with the antioxidant compound, acetyl-L-carnitine. AgNP surface modification via coating with hydrocarbons and polysaccharides led to alteration of the self-renewal and growth of mESCs. AgNP-exposed mESCs showed cell cycle arrest at G1 and S phases, which was mediated by the suppression retinoblastoma (Rb) protein phosphorylation [176]. Moreover, AgNP treatment resulted in downregulation of the expression of the pluripotency-related gene, *OCT4A* and upregulation of stem cell stress responsive genes, *OCT4B-164*, *OCT4B-190*, and *OCT4B-265*. Additionally, high ROS levels were detected upon AgNP treatment; however, polysaccharide-coated AgNPs showed low ROS production, and consequently, low toxicity [176]. Another interesting study examined the toxicity of 30 nm AgNPs during adipogenic differentiation of hBM-MSCs [128]. In this study, AgNPs showed a significant toxicity with time and dose- increment; cells exposed to 25 and 50 μg/mL AgNPs for 24 h did not show marked toxicity.

Recently, a neurotoxicity study of AgNPs was carried out on hESC-derived neurons and astrocytes and toxic effects and morphological changes were detected in the astrocytes upon exposure to high doses of AgNPs (5.0 µg/mL) [177]. Co-treatment with an antioxidant, such as ascorbic acid, abolished the neurotoxic action of AgNPs, confirming the key role of oxidative stress in AgNP-mediated neurotoxicity. In addition, the Akt/glycogen synthase kinase-3/caspase-3 signaling pathway was activated upon exposure to high dose of AgNPs. On the other hand, low concentration of AgNPs (0.1 µg/mL) boosted the ratio of astrocytes to neurons. The toxicity of the metallic NPs was concentration-dependent; further investigations are required prior to their application in the biomedical field. The toxic effect of AgNPs of size 80 nm on the osteogenic and adipogenic differentiation of hMSCs was shown in the previous report [178].

A previous study has shown the impact of AuNP and AgNP on the proliferation of human embryonic neural precursor cells [179]. In this study, 20 nm AuNP and 80 nm AgNPs were tested to reveal that AgNP-exposed cells showed apoptotic changes and high concentrations of AuNP led to a marked decrease in cell proliferation. Therefore, more detailed studies are needed for further characterization of the toxic action of the metallic NP in the nervous system.

The negative influence of TiO_2_ NPs on the proliferation of MSCs was also reported [180]. TiO_2_ NPs showed a toxic effect in MSCs in a size-dependent manner, which was highlighted by low cell migration, lack of cell membrane integrity, and suppression of the osteogenic differentiation [180]. TiO_2_ nanotubes larger than 50 nm showed a drastic decrease in the proliferation and differentiation of MSCs [139]. TiO_2_–COOH nanorods impeded the osteogenic differentiation of rBM-MSCs that contributed to the induction of the expression level of fibroblast growth factor (FGF-2) and transforming growth factor beta 1 (TGF-β1) [109].

The frequent exposure to copper oxide NPs (CuO NPs), which are found within contraceptive devices, semiconductors, and heat transfer liquids, prompted scientific interest in studying the cytotoxicity of CuO NPs. CuO NPs showed a toxic effect in rBM-MSCs in a dose-dependent manner [181]. Moreover, the surface chemistry revealed a small impact in CuO NPs toxicity. Another interesting research report evaluated the toxic effect of CuO NPs on hBM-MSCs compared with CuO microparticles. In this study, the cytotoxicity was estimated based on metabolomics [182]; an increase of glutamine was found in CuO NP-exposed hBM-MSCs, whereas high levels of succinic acid, glyceric acid, and serine were found in CuO microparticle treated cells. 

**Figure 5:**
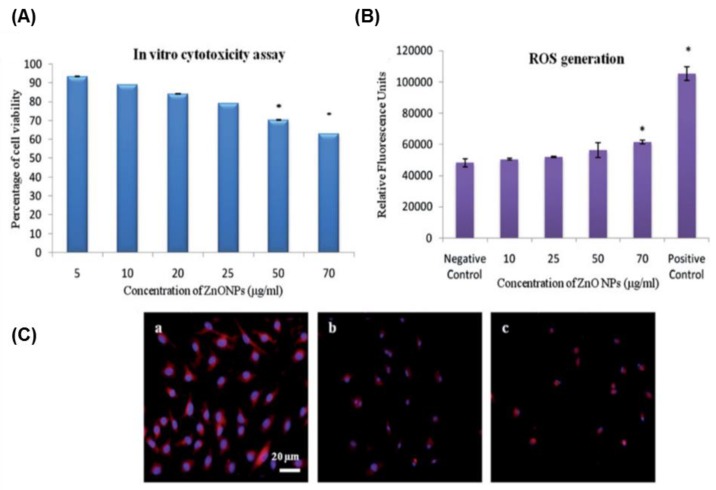
ZnO NP-induced toxicity in mBM-MSCs in ROS-mediated mechanism. (**A**) MTT assay data showing dose-dependent cytotoxicity in MSC (* *p* < 0.05). (**B**) The quantitative estimation of ROS generation in ZnO NP-exposed MSCs represented in fluorescence units (* *p* < 0.05). (**C**) ZnO NP-treated MSCs show abnormal actin filaments. Reproduced from [185] with permission from Taylor & Francis.

The impact of the size and concentration of ZnO NP exposure on the toxicity of NSCs was investigated [183]. ZnO NP-associated toxicity of NSCs was aggravated in a concentration-dependent manner (due to the release of the Zn ions in the culture medium), whereas the size did not show a marked influence on ZnO NP toxicity [183]. The influence of the exposure time of ZnO NPs on the toxicity of hMSC was investigated [184]. In this study, exposure to high concentrations of ZnO NP and repetitive exposure to low doses led to ZnO NP-related toxicity in hMSCs, which led to the cellular accumulation of ZnO NP [184]. ZnO NP-exposed mouse BM-MSCs (mBM-MSCs) showed a dose-dependent toxic action, which was attributed to high ROS production and the consequent activation of the apoptotic factors, namely caspase-3 and caspase-7 (Figure 5) [185].

**Figure 6:**
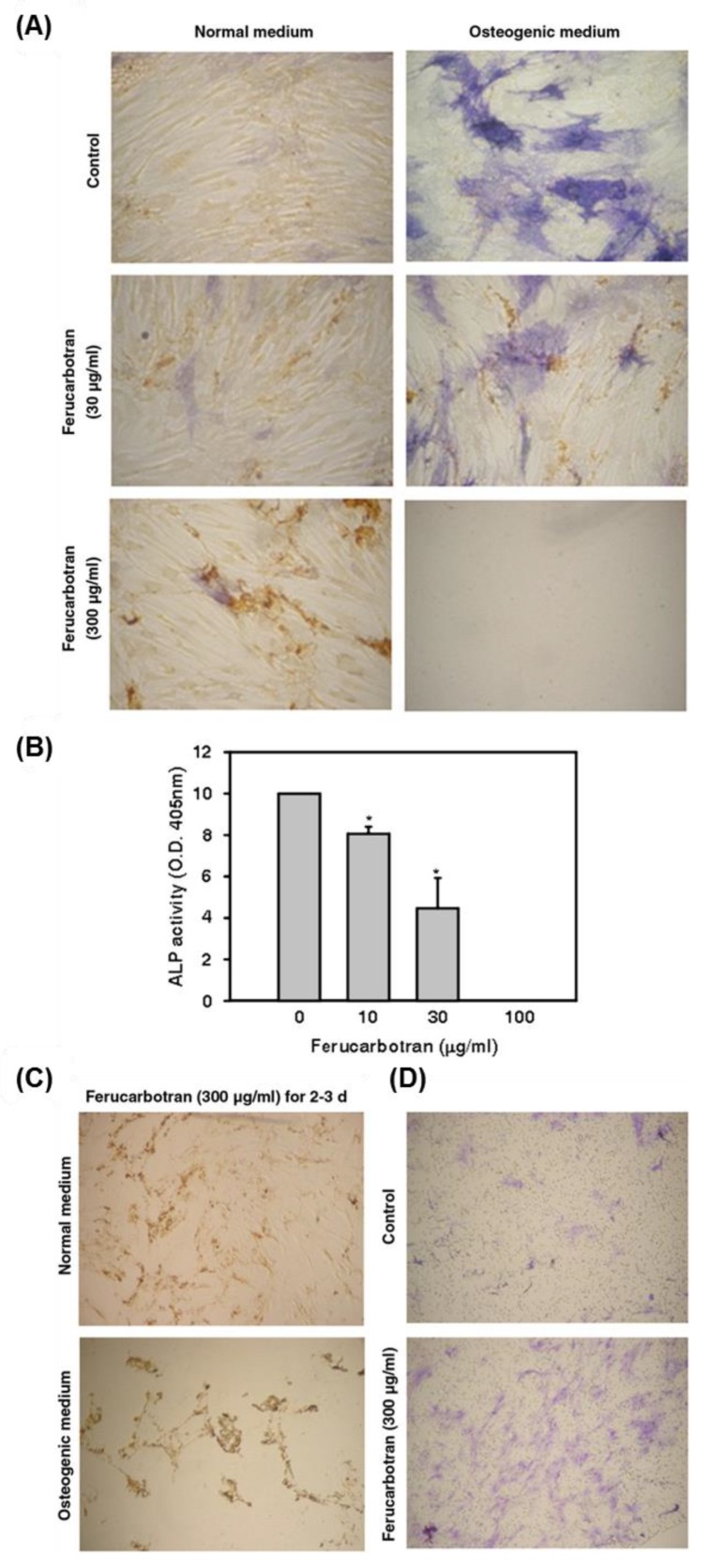
hMSC labeled with Ferucarbotran showed a dose-dependent suppression of the osteogenic differentiation and high cell migration. (**A**) Ferucarbotran (300 μg/mL) suppressed the osteogenic differentiation of hMSC that shown in weak ALP staining. (**B**) The quantitative analysis of the ALP using the microplate reader and the absorbance was taken at 405 nm (* *p* < 0.05). (**B**) The quantitative estimation of ROS generation in ZnO NP-exposed MSCs represented in fluorescence units (* *p* < 0.05). (**C**) hMSC exposed to Ferucarbotran (300 μg/mL) during the osteogenic differentiation for 2–3 days showed cell scattering and suspension, while cells adhered to the culture plate without migration under normal medium. (**D**) Cell migration assay was carried out using Transwell filters, showing the increased migration of Ferucarbotran-exposed cells for 24 h to the lower chamber that validated by the crystal violet staining. Reproduced from [188] with permission from Elsevier.

Generally, the special physico-chemical properties of SPIO NPs, such as large surface area and the enhanced reactivity of its surface, allow their transmission across the biological membranes and lead to cytotoxicity via the interaction with the cellular components such as DNA, nucleus, and the mitochondria [186]. The surface chemistry alteration of IONP via capping with citrate significantly hampered osteogenic differentiation of MSCs, as indicated by the suppression of calcium deposition and the downregulation of osteogenic differentiation-related genes, COL I and OCN [187]. In contrast, IONPs coated with pristine showed no significant suppression of osteogenic differentiation of rMSCs [187]. SPIO NPs (Ferucarbotran) showed a concentration-dependent suppression to the osteogenic differentiation of hMSCs (Figure 6) [188]. High concentration of SPIO NPs (300 μg/mL) abrogated the osteogenic differentiation and enhanced the cell migration, which was mediated via the activation of β-catenin, matrix metalloproteinase 2 (MMP2), and cancer/testis antigen, SSX. This inhibitory action was recovered with the treatment of the iron-chelating agent, desferrioxamin. Therefore, free iron is implicated in SPIO NP-induced inhibition of the osteogenic differentiation of hMSCs. On the other hand, SPIO (Feridex)-labeled hBM-MSCs showed no alteration in the cell proliferation, and osteogenic or adipogenic differentiations, but led to the suppression of the chondrogenic differentiation [189]. Taken together, the studies on the toxicity of IONPs or SPIO NPs in stem cell are obscure and need further elucidation.

Collectively, the impact of the metallic NPs on the proliferation and differentiation of various stem cells are summarized in Table 1.

## 6. Conclusions

In this review, we attempted to briefly discuss the biomedical application of metallic NPs in cancer therapy, drug delivery, imaging, antimicrobial, and cell tracking. The communication of the NPs with cellular components was also discussed. Nanomaterials possess unique properties that enable them to interact with cell membranes via various mechanisms and to be internalized within the nucleus, leading to the specific activation of crucial transcription factors and signaling pathway-associated molecules. However, further studies of the molecular mechanisms involved in NP-stem cell communication and the cellular retention and release of NPs are needed. In addition, efficient methods for the quantification of NP-stem cell interaction will need to be developed. Recently, extensive research efforts have been directed towards the application of nanotechnology in stem cell research, and several nanomaterials showed high potential for the application of stem cells in disease therapy.

Here, we discussed how the metallic NPs had a marked influence on stem cell proliferation and differentiation via various modes of action. In addition, the physical and chemical features of the NPs, as well as their functionalization were implicated in NP-mediated stem cell differentiation and proliferation. Of note, metallic NPs showed differential activity towards stem cell differentiation based on the exposure dose and time, as certain NPs promoted differentiation, while others suppressed differentiation. Accordingly, further research will be needed to determine the optimal NP exposure conditions required to obtain appropriate differentiation of stem cells. Moreover, the mechanisms behind metallic NP-based modulations of stem cell function have not yet been completely characterized, and the contradictory data on the effects of certain metallic NPs on stem cell differentiation will need to be explored further. Furthermore, the potent in vitro effects of metallic NPs on stem cell differentiation will need to be investigated through in vivo studies. There is a dearth of studies related to the impact of metallic NPs on iPSC reprogramming and differentiation, and this research will be of particular interest.

We also shed light on the limitations of metallic NPs in stem cell biology, primarily related to NP-mediated toxicity. Unfortunately, several metallic NPs have shown a negative impact on stem cell proliferation and differentiation, which is a concern that should not be overlooked. Therefore, future studies to elucidate the mechanisms behind metallic NP-related stem cell toxicity are necessary.

Many controversial studies of the toxicities on certain metallic NPs, IONPs, or SPIO NPs that need further validation. Moreover, the in vitro activities of several metallic NPs need to be confirmed in vivo. The hybrids of the metallic NPs with GO was used to efficiently monitored the neuronal differentiation status of the NSC. In addition the hybrid of SiNPs and GO markedly promoted the neuronal differentiation and the axonal growth of NSCs. However, the mechanism implicated in this effect need further in-depth studies. Moreover, novel hybrids of the metallic NPs with other nanomaterials need to be developed for promoting the high-quality stem cell differentiation and for further application in the clinics. 

In sum, metallic NPs are capable of modulating stem cell differentiation and proliferation, which highlights their potential for stem cell applications. However, further efforts are needed to find the best design and optimal exposure conditions of metallic NPs in order to obtain good quality differentiated stem cells with minimal toxicity, which will allow the innovation of stem cell therapies for incurable human diseases.

## Figures and Tables

**Table 1:** The effects of metallic NPs on stem cell differentiation, proliferation and toxicity

Cellular Effect		Nanomaterial (Name/Size)	Effect	Cell Type	Mechanism	References
Differentiation	Neural	AuNP	Enhance	mESC	Targeting mTOR/p70S6K signaling pathway	[112]
		AuNP	Enhance	F9 teratocarcinoma stem cells	-Upregulation of, RA binding protein, collagen type IV, Gata 6 & Laminin 1	[114]
		AgNP	Enhance	F9 teratocarcinoma stem cells	Upregulation of the expression levels of neural-specific markers	[133]
		DEX-IONP	Enhance	hMSCs	-DA-like neurons differentiation -Enhance the paracrine action	[153]
		IONP/HSA	Enhance	hBM-MSCs	-Covalent conjugation to FGF2 -Upregulation of MAP2 and GFAP expressions	[157]
		SiNP	Enhance	miPSC	Co-delivery of pNurr1 and siRex1	[166]
	Osteogenic	Nanoceria	Suppress	Neural stem cells	Suppression the expression levels of βIII-tubulin and GFAP genes	[173]
		AuNP (70 nm)	Enhance	hMSC	YAP activity regulation	[101]
		AuNP (30 & 50 nm)	Enhance	hADSC	Increase ALP activity	[102]
		AuNP	Enhance	hADSC	Wnt/β-catenin signaling pathway	[117]
		AuNP	Enhance	hADSC	ERK/MAPK signaling pathway	[118]
		AuNP	Enhance	hBM-MSC	Delivery of miR-29b	[120]
		AuNP	Enhance	rBM-MSCs	MAPK/p38 pathway activation	[121]
		AuNP (4 nm)	Suppress	hBM-MSC	Increase ROS generation	[190]
Differentiation	Osteogenic	AgNP	Enhance	Human Urine-derived stem cells	-RhoA activation -Cytoskeleton tension -Actin polymerization	[130]
		AgNP	Enhance	mMSC	TGF-β/BMP signaling activation	[105]
		AgNP (80 nm)	Suppress	hMSC	Agglomeration in endo-lysosomal cell compartment	[178]
		AgNP	Enhance	hADSC	Delivery of photo-activated miR-148b mimic	[131]
		AgNP	Suppress	hBM-MSCs	Enhance ROS generation	[132]
		TiO_2_	Enhance	rBM-MSC	Promote cell adhesion and spreading	[139]
		TiO_2_	Enhance	Human pulp- and adipose tissue-derived stem cells	Enhance the expression levels of bone-related genes RUNX2, FOSL1, and SPP1	[140]
		TiO_2_–COOH NRs	Suppress	rBM-MSCs	Upregulation of the expression level of FGF-2 and TGF-β1	[109]
		TiO_2_	Enhance	hBM-MSCs	High phosphorylation of FAK-mediated cell adhesion	[141]
		TiO_2_ nanotube (70 nm)	Enhance	hASCs	Promote the methylation of the histone H3 at lysine 4 in the promoter regions of the osteogenic differentiation markers	[142]
		TNT-TiO_2_	Enhance	hBM-MSCs	High surface area and the photocatalysis	[143]
		TiO_2_ nanotube (100 nm)	Enhance	rBM-MSC	Activation of MAP3K11, Na^+^/K^+^ transporting ATPases ATP1A2, and ATP1A3	[144]
		TiO_2_	Enhance	rBM-MSC	Activation of the motogenic response of MSC and release of BMP2	[145]
Differentiation	Osteogenic	IONPs	Enhance	hBM-MSCs	Activation of MAPK signaling	[154]
		IONPs	Enhance	Primary mouse bone marrow cells	Magentic field-mediated osteogenic induction	[155]
		IONPs	Enhance	hBM-MSCs	Upregulation of long noncoding RNA *INZEB2*	[156]
		Citrate-capped IONPs	Suppress	rBM-MSC	-Suppression of calcium deposition -Downregulation the expression levels of collagen type I and osteocalcin	[187]
		Fe_3_O_4_/BSA-loaded IONP	Enhance	rBM-MSC	Static magnetic field-mediated particle uptake and activation of osteogenic differentiation	[110]
		IONP/HSA	Enhance	hBM-MSCs	Covalent conjugation to FGF2	[157]
		PES-PEG electrospun composites coated with Zn_2_SiO_4_ bioceramic NPs	Enhance	hMSC	-Promotion of cell proliferation -Upregulation of ALP and the osteogenesis marker	[160]
		SiNPs	Enhance	hMSC	Activation of ALP	[162]
		SiNPs	Enhance	hADSCs	Phosphorylation of ERK1/2	[163]
		Nanoceria	Enhance	rBM-MSC	Activation of BMP signaling	[164]
		Glycol–chitosan-coated barium titanate NPs	Enhance	rMSC	-Significant increase of hydroxyapatite deposit formation -Rearrangement of f-actin based structure	[174]
		SPIO NPs	Suppress	hMSCs	High release of the free iron	[188]
Differentiation	Adipogenic	AuNP (4 nm)	Enhance	hBM-MSCs	Increase ROS generation	[190]
		AgNP (80 nm)	Supress	hMSC	Agglomeration in endo-lysosomal cell compartment	[178]
		AgNP	Enhance	hBM-MSCs	Increase of intracellular ROS	[132]
		IONP/HSA	Enhance	hBM-MSCs	Covalent conjugation to FGF2	[157]
		SiNP-conjugated insulin	Enhance	rBM-MSC	Insulin delivery	[165]
		Nanoceria	Suppress	rBM-MSC	Suppression of ROS generation	[169]
		Glycol–chitosan-coated barium titanate NPs	Enhance	rMSC	Cytoskeleton organization	[174]
	Cardiogenic	AuNP (16 nm)	Enhance	hBM-MSCs	-Formation of contractile proteins -upregulation of the cardiogenic differentiation markers	[123]
		IONPs	Enhance	hBM-MSCs	Enhance the link between MSC and the cardiomyblast via activation of connexin-43	[158]
		SiNP (50–120 nm)	Enhance	hMSC	-High focal adhesion and upregulation of connexin-43 -Promote the interaction of hMSC with cardiac myoblasts in ischemic condition	[164]
		AuNP-loaded BSA/PVA scaffolds	Enhance	hBM-MSCs	-Increase cell proliferation -Upregulation of cardiomyocyte-related protein markers	[124]
Differentiation	Myogenic	IONPs	Enhance	ESCs	Upregulation of MyoG and Myh2	[152]
	Angiogenic	Nanoceria	Enhance	Murine MSC	Upregulation of the expression of angiogenic factor VEGF	[171]
Proliferation		TiO_2_ (> 50 nm)	Suppress	rBM-MSC	Activation of the programmed cell death	[139]
		SiNPs (50–120 nm)	Enhance	hADSCs	Increase the phosphorylation of ERK1/2 signaling	[163]
		Nanoceria	Enhance	rBM-MSC	Activation of BMP signaling	[168]
		Citrate-stabilized nanoceria	Enhance	hMSC	Enhance the transcription level for mRNA of proliferation- and cell cycle-associated genes	[170]
		Nanoceria	Enhance	Human dental pulp-derived MSCs	Modulation of proliferation- and cell cycle- related gene expression	[172]
		AgNP	Suppress	Human- and rat-derived embryonic NSCs	-High ROS generation -Mitochondrial dysfunction -Activation of BAX protein -Release of the lactate dehydrogenase	[175]
		AgNP	Suppress	mESC	Cell cycle arrest via inhibition of the phosphorylation of the retinoblastoma protein	[176]
		SPIO NPs	Enhance	hMSCs	Upregulation of the cell cycle related proteins including cyclin B, cyclin D1, and CDK4	[159]
**Proliferation**		AgNP	Suppress	hESC-derived neuron and astrocyte	-Activation of Akt/glycogen synthase kinase-3/caspase-3 signaling -High ROS generation	[177]
		AgNP	Suppress	human embryonic neural precursor cells	Activation of apoptosis	[179]
		TiO_2_	Suppress	rBM-MSC	Negative impacts of cell membrane integrity and cytoskeleton	[180]
		CuO NPs	Suppress	rBM-MSC	High ROS generation	[181]
		CuO NPs	Suppress	hBM-MSCs	Upregulation of Serine, glyceric acid, and succinic	[182]
		ZnO NP	Suppress	mNSC	Inhibit mitochondrial respiration	[183]
		ZnO NP	Suppress	mBM-MSCs	-High ROS production -Activation of the apoptotic factors,	[185]

## Abbreviations

ESCs	Embryonic stem cells
ASCs	Adult stem cells
iPSCs	Induced pluripotent stem cells
MSC	Mesenchymal stem cells
SPR	Surface plasmon resonances
NP	Nanoparticle
TiO_2_	Titanium oxide
IONPs	Iron oxide NPs
AgNP	Silver NP
AuNP	Gold NP
NIR	Near infrared
SPIO	Superparamagnetic iron oxide
Nanoceria	Cerium oxide NPs
CuO NPs	Copper oxide NPs
DOX	Doxorubicin
ROS	Reactive oxygen species
ZnO	Zinc oxide
CuS	Copper sulfide
E. coli	Escherichia coli
SiNPs	Silicate NPs
FACS	Fluorescence-activated cell sorting
VEGF	Vascular endothelial growth factor
TEM	Transmission electron microscopy
ICP-MS	Inductively coupled plasma mass spectrometry
DA	Dopaminergic
RA	Retinoic acid
ALP	Alkaline phosphatase
OPN	Osteopontin
OCN	Osteocalcin
RUNX2	Runt-related transcription factor 2
COL I	Collagen type I
BSA	Bovine serum albumin
PVA	Polyvinyl alcohol
AgNO_3_	Silver nitrate
TGF-β	Transforming growth factor-beta
BMP	Bone morphogenic protein
hASCs	Human autologous adipose derived mesenchymal stromal/stem cells
CSC	Cancer stem cell
FGF-2	Fibroblast growth factor
FAK	Focal adhesion kinase
Rb	Retinoblastoma
RBP2	Retinoblastoma binding protein 2
TNTs	Titania nanotubes
MAP3K11	Mitogen-activated protein kinase kinase kinase 11
ATP1A2	Alpha 2 polypeptide
ATP1A3	Alpha 3 polypeptide
siRNA	Small interference RNA
DEX	Dextran
HSA	Human serum albumin
PES-PEG	Polyethersulfone-polyethylene glycol
PEI	Polyethyleneimine
GFAP	Glial fibrillary acidic protein
ERK	Extracellular signal–regulated kinase
MAPK	Mitogen-activated protein kinase
YAP	Yes-associated protein
6-OHDA	6-hydroxydopamine
SNpc	Substantia nigra pars compacta
TH	Tyrosine hydroxylase
BMP2	Bone morphogenetic protein 2
OMD	Osteomodulin
FOXO1	Forkhead box protein O1
ATF4	Activating transcription factor 4
MCAM	Melanoma cell adhesion molecule
CDK4	Cyclin-dependent kinase 4
ENG	Endoglin
CLCF	Cardiotrophin-like cytokine factor 1
TPM1	Tropomyosin 1
FGFR1	Fibroblast growth factor receptor 1
KRAS	Kirsten rat sarcoma viral oncogene homolog
RPS6KA1	Ribosomal protein S6 kinase, 90 kDa, polypeptide 1
CXCR4	Chemokine receptor type 4
EGFR	Epidermal growth factor receptor
GAP43	growth associated protein 43
MMP2	Matrix metalloproteinase 2
PCR	polymerase chain reaction
GO	Graphene oxide
